# Millennium Development Goals: how public health professionals perceive the achievement of MDGs

**DOI:** 10.3402/gha.v7.24352

**Published:** 2014-09-22

**Authors:** Marta Lomazzi, Ulrich Laaser, Mareike Theisling, Leticia Tapia, Bettina Borisch

**Affiliations:** 1 World Federation of Public Health Associations, University of Geneva, Geneva, Switzerland; 2 Institute of Global Health, University of Geneva, Geneva, Switzerland; 3 Faculty of Health Sciences, Section of International Public Health, University of Bielefeld, Bielefeld, Germany

**Keywords:** Millennium Development Goals, public health professionals’ opinion, global survey, politics, governance

## Abstract

**Background:**

There have been various consultations on the Millennium Development Goals (MDGs) by different groups. However, even if it is clear that the health sector has led the development success of the MDGs, only a few MDG reports consider public health experts’ points of view and these are mainly government driven.

**Designs:**

The World Federation of Public Health Associations (WFPHA) has executed a global survey to consult public health professionals worldwide concerning the implementation and achievements of the MDGs.

The survey was conceived by WFPHA health professionals and promulgated online. Public health professionals and organisations dealing with MDGs responded to the survey. Content analysis was conducted to analyse the data.

**Results:**

Survey participants attributed the highest importance worldwide to MDGs dealing with women, poverty and hunger reduction, and disease prevention and management. Moreover, they underlined the role of education, referring both to school children and professionals. In high and upper-middle income countries, environmental challenges also received considerable attention.

Notably, respondents underlined that weak governance and unstable political situations, as well as the gap between professionals and politicians, were among the main causes that detracted from MDG achievements.

**Conclusion:**

The public health workforce felt it would be imperative to be included from the outset in the design and implementation of further goals. This implies that those professionals have to take an active part in the political process leading to a new and accountable framework.

The Millennium Development Goals (MDGs) are eight global targets facing poverty, hunger, maternal and child health, communicable disease, education, gender inequality, environmental decay, and the global partnership (Table 1); they have been formally established as an output of the United Nations Millennium Declaration, following the Millennium Summit of the United Nations in 2000 (1, 2). All 189 United Nations member states agreed on a voluntary basis to attain these goals by the year 2015. New international health initiatives and increased available funding have facilitated the development of MDGs-related programs worldwide (1, 3).

**Table 1:** The eight Millennium Development Goals

MDG1	Eradicating extreme poverty and hunger
MDG2	Achieving universal primary education
MDG3	Promoting gender equality and empowering women
MDG4	Reducing child mortality rates
MDG5	Improving maternal health
MDG6	Combating HIV/AIDS, malaria, and other diseases
MDG7	Ensuring environmental sustainability
MDG8	Developing a global partnership for development

General broad agreement has been reached on the basic importance of the MDGs as a catalysing element in the global agenda and as a force for maintaining political support for development (4, 5). Until now, several targets have been at least partially achieved: hunger reduction is progressing as planned, poverty has been halved, the living conditions of 200 million poor people enhanced, maternal and particularly child mortality as well as communicable diseases have been reduced to a certain extent and education improved (6). However, in spite of the general positive outputs, these global targets, as well as the framework adopted have often been criticised (e.g. due to the untied nature of the goals, the weak link with sustainable development, the limited focus on equity, the unequal level of achievement between and within countries) (7–13).

There have been several consultations on the MDGs by a number of organisations. Some of the consultations and surveys have been officially led by governments while others can be considered ‘private’ initiatives, driven by non-governmental organisations (NGOs) and private foundations (1, 14–19). Numerous official reports have traced the global assessment of progress, based on various data (5, 16, 20, 21).

However, even if it is clear that the health sector has played a major role in the achievement of the MDGs (12, 22), only a few MDGs reports have considered public health experts’ points of view and the role of public health itself (21). In this sector, public health professionals have an important role to play and are often placed at the interface between political and medical decisions. In this context, public health is the driving force for the implementation of certain MDGs. Thus, public health professionals’ perspectives are of primary importance for obtaining a complete and more objective overview of the MDGs achievements and failures.

The World Federation of Public Health Associations (WFPHA) (23), in its role as the umbrella organisation of Public Health Associations and professionals, has decided to collect the feeling and opinion of professionals involved on a daily basis with MDGs-related activities and to give voice to this constituency. Professionals’ fields of activity range from grass-roots workers to senior civil servants, generating views that come from diverse standpoints.

## Methods

The global survey (available as on-line supplementary material) was developed by WFPHA health professionals in association with the WFPHA Equity Working Group between January and March 2012 and subsequently passed by the WFPHA governing council. The survey was translated into four languages (French, Spanish, Portuguese, and Chinese) from the original English version and promulgated online through www.surveymonkey.com. We invited all public health professionals and organisations (*n*=5,014) listed in the WFPHA database by e-mail between April and July 2012 to complete the survey if they were involved in MDGs-related activities. We also advertised the survey in the WFPHA newsletter, Facebook page, Twitter and during the 13th World Congress of Public Health (24).

We received 427 completed questionnaires, representing professionals from 71 countries, covering all WHO regions [detailed description available in ref. (16)]. Both quantitative and qualitative data have been collected through the survey (questions used to collect qualitative data are reported in Table 2).

**Table 2:** Questions used to collect qualitative data

Q13: Why are the MDGs selected above of highest importance for your country?
Q24: Briefly describe the main MDG-related activity you/your Public Health Association/Organisation undertook (including major achievements and failures)
Q28: How did these obstacles affect the achievement of the targeted MDGs?
Q30: Please briefly describe this on-going activity
Q32: Would you like to add your PERSONAL point of view/feelings regarding MDGs projects/achievements/challenges in your country?
Q33: Would you like to add any comments/suggestions?

The quantitative data has been published separately; please refer to this publication for all quantitative results (i.e. respondents’ positions, regional distribution, etc.) (16). The qualitative data are reported in this article.

Inductive content analysis was conducted (25). Only answers from public health professionals directly involved in activities related to the MDGs have been retained. The statements were analysed in their original language except for those in Chinese, which were first translated into English. Answers were transcribed and coded. Firstly, each answer was reviewed line by line by two authors independently and coded according to the main subject categories. Subsequently, similar categories were grouped into themes (see Table 3). A third expert reviewed the final categorisation. The qualitative data analysis package MaxQData was used for the initial stages of coding (26). However, the software was used as an aid for organising the material and not for interpretation.

**Table 3:** Categories and themes (in alphabetical order of themes)

Themes	Categories
Advocacy	Advocacy and policy development Amplification citizens voice Improving evidence-based decision making Lobbing Technical support to government
Behavioural changes	Individual/community awareness of MDGs Individual/community acceptance of changes necessary to achieve the MDGs Religious and cultural barriers
Community empowerment	Individual/community empowerment Health promotion and education within the community
Disease prevention and management	Communicable diseases Non-communicable diseases Emerging diseases
Economy	Resources available Donors engagement Use of resources Economic and financial crises Economic development
Education	Education (general) Education for non-professionals/primary education Education for public health professionals Information/knowledge exchange (IT tools and social media)
Environment	Sustainable development Environment health
Governments	Influence of authorities MGDs funding selection Corruption (use of money and false reports) Coherent engagement of governments
Health system strengthening	Infrastructures and services availability, accessibility, and utilisation Primary health care Strengthening of infrastructures and services
MDG project management	Research Development Collaboration Fundraising Monitoring and evaluation Implementation
Post-2015	New agenda Research (stakeholders involvement)
Poverty and hunger	Poverty Hunger
Public health workforce	Public health workforce availability Public health workforce motivation
Women	Maternal and child health Inequality/gender

We firstly analysed all results together, and then separately according to respondents’ roles (individual professionals versus official spokespersons of public health associations), WHO regions, main countries, and country's Gross National Income (GNI) according to the World Bank Indicators (High Income Countries – HIC; Upper–Middle Income Countries – UMIC; Lower–Middle Income Countries – LMIC; and Low Income Countries – LIC) (27).

The results originate from inductive qualitative analyses with the support of the MaxQData program that enables the counting of the number of answers dealing with a specific category/theme (defined through the inductive analysis), thus rating the categories/themes most cited; this approach was adopted to analyse all results together as well as separately according to respondents’ roles, WHO regions, main countries, and GNI (26). The main percentage agreement (28) is reported in the results.

A selection of the most relevant full respondent statements, selected from 1,769 statements (translated to English, and slightly edited when required) is reported in the text. The statements selection has been conducted according to the ‘Guidelines for Critical Review Form: Qualitative Studies’ by three authors independently to reduce the bias and give the most complete overview; however, the researcher's bias and influence of the researchers’ point of view cannot be completely avoided as per the methodology itself (29).

## Results

Here, we report the main qualitative results, including sentences and main percentage agreement. Quantitative data have been published separately (16).

We describe respondent's opinions all together and classified according to their regional importance where relevant. Indeed, the local importance of MDGs was truly different between regions and countries in the world and also depended on whether the public health professionals came from a high or low/middle income country. Major regional differences were observed in association with the quantitative data and have been published elsewhere (16).

Firstly, respondents attributed the highest importance to MDGs dealing with women, poverty and hunger reduction, disease prevention and management (percentage agreement: 34, 20, and 19%, respectively), and emphasised the interdependency of these goals.

Participants knew and understood the importance of integrating gender perspectives in all health policies. They recognised that improvements in women's living conditions, that is, better maternal (and child) health as well as women's empowerment together with clear inequality reduction, represented the most cost-effective achievement for health systems. Indeed, if this target were attained, it would enable not only better conditions for women but also improved household management, leading to better health and education for children and to higher income for the families.

Improving maternal health means reducing child mortality (i.e. breastfeeding), improving gender equality and women empowerment means that women can work and share motivation and ideas … means achieving universal primary education since education relies first on mothers … means eradicate extreme poverty and hunger; when keeping mothers healthy, each mother can work hard creating incomes and giving ideas … means keeping family health since mothers keep the family clean from dirty things reducing transmission of diseases; mothers take their child to vaccine station in proper time and are willing to take advice from the health professionals

… more women are in politics (and more) things get better; majority of African women do things perfectly fine despite injustice and bribe

The same interdependency observed between women and health was also attributed to poverty reduction or disease prevention and management and health. Participants recognised that eradicating extreme poverty would lead to improved living conditions for individuals, family units, communities, and countries; as a consequence, more and better health services would be available and easier to reach, allowing for improved prevention and management of diseases. Healthier people would be able to work more, increasing their personal income and their country's wealth; which would mean more money to invest in improving the quality of life at multiple levels.

A hungry country cannot develop, that's why MDG1 is so significant to the Gambia

South Africa is in the midst of an AIDS pandemic that is affecting most people in one way or another, and will have long term consequences on the economy

Poverty is the cause of ignorance, child and maternal mortality, and environmental degradation

Secondly, public health professionals claimed their role in the debate on MDGs and in the definition of the post-2015 agenda. Participants suggested making decisions on a solid basis, which means including public health stakeholders in the discussion and taking advantage of objective reports that are not government-driven. At this level, policy development and advocacy activities play a primary role in increasing leader awareness and engagement.

Personally I feel that there is a huge gap between professionals experience and attitude which mainly contradicts with politicians. … it would have been better focusing on the real achievement by directly utilising experts

The government must do a rethink; go back to the drawing board with the right professionals! The intra-professional discrimination should give way to liberal broad recognition of Public Health Physicians

Discussions on the MDGs at the international level often left out many of the stakeholders most effective in those areas. The lack of progress in MDG5 and to some extent for MDG1 and MDG6 could have been mitigated with more inclusive involvement

Thirdly, remarkable importance was attributed to ‘politics’. The in-depth analysis of the text answers showed that this heading may have covered points ranging from low political commitments to corruption and nepotism as well as to organisational aspects of both the political and health system. Respondents underlined the need for prioritisation and for a more operational choice of MDGs, less influenced by global politics, and adjusted to the capacities of nations, especially for developing countries. Donor nations should instead focus on sustainable initiatives aimed at the development of effective global partnerships.

There are several challenges and factors contributing to the failures of MDGs achievement: large scale corruption at each level, bureaucratic system of organisations and nepotism and parochial practices

I believe not controlling but at least reducing corruption is one step ahead for achieving MDGs

(MDGs have not be fully achieved) due to permissiveness and inertia of the (public) administration plagued by corruption which limited all efforts for development; this was due to an insufficient political willingness, lack of good governance and freedom of authorities due to the regime in place that refuses all political changes that could boost the development and increase population trust

Governments (and donors in general) were also criticised for the reduced funding allocated to the MDGs as a consequence of the economic crisis, and for the lack of in-country resources made available. They recognised that these challenges had deeply affected project management, leading to delays and problematic project development, monitoring, evaluation, and implementation. The limited resources available, as well as the poor quality of services, had not allowed achieving all goals in a sustainable way or reaching all areas, leaving out marginalised populations.

The global economic crisis has seriously affected the capacity to implement project activities in the areas they were supposed to scale up and make meaningful contributions

To best utilise the limited resources available, an effective monitoring and evaluation of the whole process as well as improved and more formalised coordination between the different actors (governments, international communities, local associations, etc.) was recommended.

In many remote/rural areas of Nigeria there are a beautiful new primary health care (structures) built with MDGs money which are empty and unused except for chickens

For Australia, as a donor nation, we must ensure that our ODA is sustainably given (i.e. it builds capacity, empowering people to ‘do for’ themselves, rather than doing it for them) and that it is also environmentally sustainable (i.e. supports projects that enhance, rather than detract from, the wider biosphere)

International collaborations should be strengthened, funds should be materialised (instead of financial), and control should be made on its utilisation

Fourthly, survey participants underlined the importance of projects dealing with education referring both to school children and professionals.

The lack of individual and community empowerment was often cited among the main problems for the goals achievement in LMICs and UMICs. Primary education was described as the base for achieving this empowerment, especially in regions where religious, cultural or political barriers deeply affect people's behaviour or where unhealthy lifestyles had led to health-related problems. In addition, the gendered aspect of education and its consequences were recognised especially in sub-Saharan Africa.

Inequality is the largest challenge in reaching MDGs, especially for education

The male dominated decision making process and power is still prevalent in the conservative communities in most of sub-Saharan Africa

Education is a key strategy for all maternal death; this is a neglected tragedy

Respondents underlined that these barriers made the introduction of public health measures at the community level difficult; thus, activities aiming at increasing people's awareness and inducing behavioural changes at the community level were the foundation for the acceptance of all MDGs.

Professional education and training would lead to strengthening of the public health workforce. Respondents emphasised that public health professionals should be oriented to preventive community intervention. In the actual situation, public health workers’ energies were frequently diverted to medical care activities rather than public health actions, and this tendency should be reversed.

The main limiting factor for these activities is the strong biomedical orientation of health professionals which are neither focused on (health) promotion and prevention nor on community intervention

In addition, participants asserted that the MDGs lacked substantial visibility among public health practitioners, and that public health workers should be instructed about the MDGs concept.

Awareness of the MDGs is generally low among the general population and not a core area of concern or acknowledgement for most public health practitioners

Furthermore, respondents envisaged exchanging information and knowledge broadly through the new technologies available (i.e. on-line training, etc.).

(It is important to) organise trainings using virtual online network to reach more people; focus more on use of telephony and other ICT infrastructure (mHealth, etc.) to reach the under-served

Improving the communication around MDGs would not only allow better education but also a more effective advocacy for MDGs at all levels.

Additionally, participants underlined that not only trained but motivated public health professionals are necessary to get positive outputs. Thus, the public health profession should be recognised and employment opportunities should be offered in fair competition.

Lack of motivation (among public health professionals) increased the rate of dropout

Fifthly, in countries with better health indicators environmental challenges have received high attention (percentage agreement >20%); respondents understood that worldwide climate change has had an impact on most MDGs, that is, on the appearance and re-appearance of communicable diseases. In this context, sustainability plays a crucial role.

I fear still the risk of malaria re-emergency due to climate change and need a great concern

There is need to evolve sustainable framework for interventions targeted at achieving specific MDGs through developing in-country system for sustainability

Environmental health activities, such as water sanitation, waste, and air quality management were recognised as being of primary importance for all countries and their future development.

## Discussion

Public health professionals widely agreed that a major part of the MDGs has been at least partially accomplished and underlined the importance of women's health and empowerment, poverty reduction, disease management and control, and education in the process, in line with most official reports (1, 5, 6).

However, the MDGs will not be fully achieved due to various reasons such as the limited resources available, lack of services, and trained workforce or lack of coordination. Moreover, a single MDG will most possibly not be achieved in a country where all remaining MDGs have not been reached.

While these topics have been already largely discussed in many official reports and consultations, the most salient points that this study reveals are the role claimed by public health professionals in the global MDGs decision making process as well as the weight of ‘politics’, including aspects such as governance and corruption.

First, public health professionals claimed their role in the political debate aimed at defining and implementing the global and local goals, taking advantage of their knowledge and experience. Achieving the health-related MDGs takes a workforce. Central to this are all public health professionals working within health services or at the community level, who promote health and provide health services. Those people are responsible for the development, monitoring, and implementation of public health programmes within their communities. As a follow-up, the participation of communities and civil society is of primary importance both for strong policy development and for holding all stakeholders and politicians accountable for progress. Good governance will require coordinating a coherent response across government and society that results in better health outcomes (‘health in all policies’). In this context, public health activists should play the role of fostering networking between civil society, governments, and corporations (22, 30). Nowadays, some platforms for discussion allow civil society worldwide to express its aspirations and actively take part in the development of the post 2015 agenda (31, 32). Through these platforms, as well as other formal and informal consultations, governments, intergovernmental and multilateral organisations recognise the role of the health professionals and civil society in general and engage them in the process.

In this setting, public health experts can play their role as implementers and monitors of health-related goals worldwide and provide accountable feedback to governments; governments should effectively build on their decisions, taking into account the feedback and implement activities necessary to achieving the goals and wellbeing in general. This new role covered by the public health workforce was not recognised during the development of the MDGs, which were mainly driven by member states.

Moreover, innovative solutions and technologies can and should be provided by developed and developing countries. The north–south division is no longer applicable. There is room for south–south dialogue as well as for the south to north innovation scheme. The MDGs framework itself constitutes a concept that does not facilitate this interaction but reduces goals to a list of eight and neglects their interconnections; it encouraged sustainable pro-poor development progress and donor support but does not take into account the innovative solution that developing or in-development countries can offer (1, 33, 34). This result adds up to critics that only very few actors guided the development of the MDGs (35).

Second, in numerous MDGs surveys (12, 36) as well as in the post-2015 consultations (22), aspects of governance and corruption are less apparent even if corruption can deeply hurt health outcomes. This may be related to the form and way of the survey (37). It may also be due to the fact that other groups outside the health sector [i.e. International Association of Anti-Corruption Authorities (38)] are dealing with this problem (39), and that there are only a few links between the two communities. The anti-corruption agencies are indeed mainly dealing with anti-money laundering, criminal law enforcement, and the recovery of stolen assets. Only few studies looked at the corruption that occurred using the MDGs money itself (40). From the present answers, the need for addressing political aspects such as governance and corruption seems obvious. A health care system in a corrupt setting is weak, unsustainable, and cannot last for long; public health professionals locally know that an effective health system will be achieved only with a good governance system in place (1). One of the goals of WHO is strengthening health systems and this is widely accepted (41). It may be considered to broaden this goal to ‘strengthening governance systems’ in general and to address both the health and the governance aspects of the development agenda at the same time (1, 42). But, even in settings with a high degree of governance, health systems tend to be easily reached by corruption-like mechanisms (43).

Some studies have been run in recent years to define methods, tools, and good practices to map corruption risks, develop strategies, and partnerships to address challenges as well as block corruption in the health sector, with the final aim being to improve accountability and service delivery post-2015 and beyond (44). The costs of corruption can be explicit, implicit, and hidden; politicians must recognise these challenges and find solutions to incorporate the MDGs and anti-corruption agendas in a global health governance framework and develop protocol to combat this crucial issue (45, 46).

A final aspect that should be taken into account is the lack of confidence into the official reporting systems associated with the need for unbiased and all inclusive reports, which will also require prioritising follow-up to make sure governments implement audit recommendations (45). Accountability remains of primary importance; transparent and effective monitoring and evaluation should be guaranteed to allow a proper implementation of the activities.

Our findings should be considered in light of some limitations. First, most of the answers have been collected from public health professionals from the African and Western Pacific Regions and further research should compare the results described here with greater representation from other regions (16). However, there was substantial agreement across all regions, even if representation varied. Secondly, there might be some weaknesses in the translation from Chinese.

Despite these limitations, we think that the qualitative findings reported are of high importance and provide more insight into the real situation than the quantitative interpretation alone (16).

## Conclusions

Salient points of our study show that the public health workforce at all levels wants to take an active role in the decision making process in a constructive dialogue with politicians and other stakeholders. Moreover, public health professionals feel it most important to have good governance as an essential prerequisite to achieve population health. Together, these issues should be taken into account in the debate and definition of the next round of goals beyond 2015.
